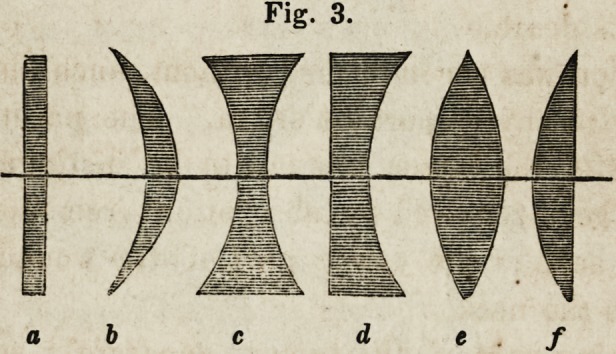# Histology—Its Importance, &c.

**Published:** 1857-10

**Authors:** 

**Affiliations:** in the College of Dentists.


### ARTICLE VII.

*Histology—Its Importance, &c.* Lecture by Mr. Hogg, M. R. C. S., in the College of Dentists.

Before I enter into the important subject matter I have been desired to lecture upon, viz. histology, I must crave your kind indulgence for many imperfections and short comings you will from time to time perceive; as what I have now prepared is intended for the tyro, and I hope you will regard it as merely introductory to a more systematic course of histological investigation. The great importance of microscopy in the study of anatomy, physiology, and pathology, has been long since conceded by the scientific and learned, among the medical and other enlightened professions, of this and every other civilized nation. I know full well, that the members of this branch of the profession have acknowledged its importance; and the microscope has been cultivated with a zeal worthy of your enlightened body; and that you have in your ranks men who have contributed much towards our knowledge of the microscopic structure, not of the teeth only, but of many other important tissues of the animal body. None here can require me to tell them that first and foremost in the rank of microscopists, stands the late Alexander Nasmyth, whose reputation, from his valuable original researches, is European. Many others might be mentioned, but they are already well known to all of you.

When I think of such men, I cannot help feeling a deep sense of the responsible task I have undertaken; and my own weakness and deficiencies have become more apparent in my attempts to deal with the subject in a way that it should be dealt with; to point or guide the student to the great and wondrous succession of phenomena, and the unbounded objects of interest, presented in nature's secret workings, not only in this *special*, but in every other department, wherever he may direct his scrutinizing eye.

Hunter complained of the ignorance that prevailed in his day of the diseases and anatomy of the teeth. To remedy the evil, he set about writing his book on the "Natural History of the Teeth," "that dentists may be sufficiently instructed in the anatomy, uses and diseases, of every part of the tooth." You will more fully comprehend his labors in the good work, when I tell you that in the Hunterian Museum, at the Royal College of Surgeons, and accessible to you all, are to be found a series of preparations of the teeth, prepared by his own hands. The series consist of 170 preparations, derived from all parts of the animal kingdom; the account of which in the catalogue is preceded by some general observations on their nature; on their analogy to the beaks of birds; and on the varieties they exhibit in number, form, structure and situation, according to the food of the animal, as also on the mode of their formation and growth; on the manner in which the place of those teeth which are lost is supplied; and on their relation to the stomach. These several points are illustrated in a number of sub-series, of which the three first exhibit the structure and growth of parts analogous to teeth in various tribes of animals, as the calcareous teeth of some mollusca, the bills of birds, and the whalebone in whales. The fourth sub-series exhibits the mode of growth of various teeth: first, of such as are limited in their growth, and which, when lost, requires to have their places supplied by new teeth; second, of such as are continuous in their growth, as the incisors of rodentia, the tusks of boars, &c.

The amount of animal matter contained in various descriptions of teeth, is shown in a set of preparations, in which the calcareous part has been removed by the use of dilute acids; the manner in which the teeth are shed, and their places supplied by new ones. The last sub-series displays the varieties in the situation of the teeth, which may be placed either on the jaws, the tongue, the palate, or in the stomach.

Such labors occupied the attention of Hunter, and many good and true men of science have followed in his steps;* and the time has now arrived when the education of gentlemen preparing to practice the dental art, will no longer be permitted to enter upon so responsible a duty, without a sufficient preliminary education. Your president and council, then, in my humble opinion, have acted most wisely in taking the step they have; and sooner or later, not only the whole profession, but the public, will acknowledge their services, by recognising and consulting only those who possess the diploma of the college of dentists.

It is within the memory of many among us, that the course of study required by the medical colleges, and the status of the whole profession, was very different compared to what it is at this time. Not many years ago, a course of twenty-three lectures was considered sufficient to qualify the medical student, and he was then admitted to practice his profession, often long before he had attained to twenty years of age. The case is much altered now; and a medical man believes himself to be a student for life: in other words, our education is never completed. I refer to this, because it has been said that your prescribed course of study is not what it should be; a very short time will correct this, as not only your council, but those who have undertaken the present course of lectures, wish you to understand, that these are merely a *preliminary course*, or as your President told you a few evenings since, *introductory*. Every one here, I am sure, believes in the importance of the study of anatomy as at present taught in our medical schools, as well as that of the various other departments of medical science, which must be looked to, for the purpose of giving your

* Nasmyth's large and valuable collection of teeth and microscopic preparations were purchased by the council of the college at his death, and have been added to the Hunterian collection.

students the necessary information on the structure, functions, and remedial agents, employed in the dental branch of the healing art. Your students will be expected, also, to become acquainted with the healthy condition of the organs, upon which depends so greatly the happiness and comfort of every one in this life. The seat and cause of derangement, or disease, will necessitate a knowledge of how to employ therapeutical agents, so as to secure their due effect; equally with the skill to use mechanical helps to restore the natural action, or preserve the natural structure of the part.

A knowledge of surgery, to a certain extent, will be found essentially useful, in the repair of defects, diseases, or injuries. Mechanics—mechanical surgery—will be of use in supplying laws and proper principles for guiding the mighty powers of the lever; the hand then becomes more skillful, and difficulties that often beset operations in the mouth, as much as other parts of the body, are readily surmounted.

Chemistry is of great importance, as by its aid we are made acquainted with the properties of every form of matter. In this is included the study of the chemical composition of the teeth, as well as of the soft parts which surround them, and the secretions which flow over them. Since the health of the mouth is essential to the perfect health of the stomach, and since diseased conditions of the latter react upon the former, it becomes us to understand the functions of the one, if we would fully comprehend that of the other. Allied to this consideration, is that, of the physiological chemistry of the natural organs of mastication, which at the present time is always associated with the study of histology,* or the study of the science of the tissues entering into the formation of the animal body, since "the general doctrines towards which the labors of microscopists are manifestly tending, in regard to the laws of organization and the nature of vital action, seems fully deserving to take rank in comprehensiveness and importance with the highest principles yet attained in physical or chemical science."

* From ιστιος, *a tissue* or *web*, and λογος, *a discourse.*

With respect to the means by which histological researches are carried out, it is requisite, in the first place, that the student should be furnished with optical instruments in which he can have full confidence, but his mode of investigation must be directed by the nature of the structure to be determined. To solve the difficulties of pathological histology, it is not alone sufficient to give a precise description of anatomical conditions as seen by the naked eye, but also of the elementary constituents, and the origin, progress, and retrogression, of the abnormal tissue, as analysed by the microscope. The microscopical examination, therefore, starts from what has been seen by the naked eye, and presupposes strict observation by the latter. It is evident that histological research of this kind must demand considerable care and attention, as well as a previous knowledge of the microscope. Of all the instruments which have been applied to scientific research, there is, perhaps, not one which has undergone such important improvements, within so brief a space of time, as the microscope has received during the last quarter of a century, and there is certainly none whose use, under its present improved form, has been more largely or more rapidly productive of valuable results. It is but proper, then, that I should first very briefly tell you something of its early history.

Was the microscope known to the ancients? Many among the learned refuse to the ancients a knowledge of magnifying glasses, since according to them the Greeks and Romans had only very imperfect notions with respect to the fabrication of glass. From a passage in Aristophanes, it is plain that globules of glass were sold at the shops of the grocers of Athens, in the time of that comic author. He speaks of them "as burning spheres." Pliny states that the immense theatre (it was capable of containing eighty thousand persons) erected at Rome, was three stories in height, and that the second of these stories was inlaid with a mosaic glass.

Ptolemy in his "Optics" has inserted a table of the refractions which light experiences under different angles of incidence; in passing from air into glass. The value of these angles, which differ only in a slight degree from those obtained in the present day by means of similar experiments, proves that the glass of the ancients differed very little from that manufactured in our own time.

There is in the French cabinet of medals a seal, said to have belonged to Michael Angelo, the fabrication of which it is said, ascends to a very remote period, and upon which fifteen figures have been engraved in a circular space of fifteen millimetres in diameter. *These figures are not all visible to the naked eye.*

Cicero makes mention of an Iliad of Homer, written upon parchment, which was comprised in a nutshell. And Pliny relates, in his "Natural History," "that Myrmecides, a Milesian, executed in ivory a square figure which a fly covered with its wing." Unless it be maintained that the powers of vision of our ancestors surpassed that of the most skillful modern artists (which could be disproved by the multitude of astronomical observations made by them,) these facts establish that the magnifying property of lenses was known to the Greeks and Romans nearly two thousand years ago.

We may besides advance a step further, and borrow from Seneca a passage whence the same truth will emerge in a manner still more direct and decisive. In the Natural Questions we read, "However small and obscure the writing may be, it appears larger and clearer when viewed through a globule of glass filled with water." Duteus says, he "has seen in the museum of Portici old lenses which had a focal length of only nine millimeters." He actually possessed one of these lenses, but of a longer focus, which was found in the ruins of Herculaneum.

At the meeting of the British Association, held at Belfast in the year 1852, Sir David Brewster showed a plate of rock crystal worked into the form of a lens which was recently found among the ruins of Nineveh, by Mr. Layard. Sir D. Brewster, so competent a judge in a question of this kind, maintained that this lens had been destined for optical purposes.

The compound microscope appears only to have been known and first constructed in the early part of the seventeenth century. Both Holland and Italy have claimed the honor of producing its inventor. Borellus attributes its construction to Zacharias Jansen, of Middleburg, in the Low Countries, who, with his son John, according to this author, made his first compound microscope so early as 1590. It is stated that either he or his son presented one of his instruments to the Archduke Charles of Austria, who, in turn, gave it to Cornelius Drebbel, a Dutch alchemist, who subsequently became astronomer to James I, of England. He it was who first brought the instrument to London in 1619, where it was seen by William Borelli and other scientific individuals. It is well known that Drebbel made microscopes in London in 1621, and generally past for their inventor.

On the other hand, Francis Fontana, a Neapolitan, states, that he invented the instrument in 1618, and gave a description of it in his "Novæ terrestriume et cælestium observationes." It would appear, however, that although Drebbel and Fontana disputed concerning the origin of this instrument, the honor of inventing it, so far as our present knowledge extends, belongs to Jansen.

The improvement of the microscope made much less rapid progress than that of the telescope. The great utility of the latter, indeed, appears to have been early appreciated, while the microscope was for a long time only regarded as a means of satisfying curiosity, or as a toy. At a later period, however, it was found susceptible of adding much to our knowledge of the natural sciences; and, no sooner was this perceived, than the most celebrated artists, mechanics, geometricians, and natural philosophers paid great attention to its improvement. For a long time, however, they were baffled by the difficulties of the undertaking, and during this period, naturalists, for the most part, employed the simple microscope. Thus, some of the most important discoveries have been made by means of a single biconvex lens, and the laborious and brilliant researches of Leuwenhoeck, Swammerdam, Lyonet, Ellis, and others were thus accomplished.

The inconveniences of the simple microscope, however, are very great. Thus, when capable of magnifying largely, the field of vision is very limited, and there is great difficulty in adjusting the focus. Leuwenhoeck had a separate lens especially adapted to one or two objects, and always had several hundreds at his disposal.

The imperfections of the compound microscope, on the other hand, were, at that time, very great, and must have appeared insurmountable. Thus, from its peculiar construction, the rays of light were readily decomposed, and circles of different colors surrounded or tinged the object, constituting the aberration of refrangibility. The form of the object was also distorted on account of the aberration of sphericity. Opaque objects could not be seen from the absence of light, and very transparent ones could not be examined from its excess.

But gradually all these different obstacles were overcome by patience and labor. The details connected with these, however, would detain us too long to enter into. Suffice it to say, that to Liberkuhn we are indebted for the means of examining opaque objects by means of a reflector; to the diaphragm of Le Baillif, for a convenient mode of modifying an excess of light. Achromatic instruments were constructed principally through the ingenuity and labors of Euler, Dolland, Frauenhoser, Selligue, Amici, Huyghens, Charles Chevalier, Lister, Brewster, and others.

But it is not enough to have good instruments; we must know how to use them. Descriptions and figures, however they may assist instruction, wholly fail in communicating just ideas of structure. To arrive at this every one must look for himself.

Thus it often happens that persons for the first time looking through the microscope, are really unable to see what is placed before them, from having first their attention directed to globules of air, pieces of dust, shreds of cloth, &c., which appear prominent in the field of the microscope, but which he who is accustomed to them entirely overlooks. It was in ignorance of these circumstances, perhaps, rather than from the badness of the instruments employed, imperfect as these undoubtedly were, that the numerous errors on the subject of structure originated among the early observers. "Hence," says Burdach,

"it may be affirmed that Monro would have come to the same conclusion that he did, even though he had had a microscope of Frauenhofer; and that, on the other hand, Ehrenberg, even with Della Torre's imperfect lenses, would have made out the structure of the nervous substance as he has now done." In short, the sense of sight as applied to the microscope, must undergo a new education, and our ideas of organized structure be completely changed, before it is possible to arrive at satisfactory results. In no case can this be accomplished without practice and industry; but here, as in other branches of knowledge, proper instruction may do much.

To point out in detail the discoveries made through the employment of this instrument, as regards physiology, would be to give a history of modern biological science; for there is no department in this study which is not more or less grounded upon the facts the microscope has revealed.

The invisible life of modern times, as disclosed by the microscope, has been the subject of careful study by the naturalist and the physiologist. All space—within us and without us, and around us—swarms with its countless millions, and on whatever speck or atom of life rest our eyes, we learn the instructive lesson, that man is not the only creature that is "fearfully and wonderfully made." But, however interesting is the study of microscopic life, and however beautiful its forms and startling its functions, the microscope claims a higher value in having given birth to the truly useful science of histology, which describes the structure of animal and vegetable tissues in reference to their origin and development. The elementary tissues of animal and vegetable life have been eagerly studied both in their structure and functions, and physiologists have been thus led to the remarkable conclusion, that each integral portion of the animal or plant possesses an independent life of its own, and is performing a series of actions peculiar to itself.

By microscopic researches, Schleiden pointed out, that in vegetables, the earliest traces of organization in the embryonal sac of the unimpregnated ovule was the appearance of minute granules, which augment in size, originate cells, and constitute the structural basis of every plant. From the different transformations these undergo, all the different tissues in vegetables are formed; for instance, the spiral and dotted ducts, woody fibre, and so on. Schwann showed that the formation of tissues in animals went through much the same process, a fact that has been confirmed by the microscopic observations of Valentin and others. Thus, vessels, glands, the brain, nerves, muscles, bones and teeth, are all formed from metamorphosed cells.

But in pathology, how vastly important, nay, how absolutely necessary, is an appeal to the microscope. How often are men, who have passed their lives in the examination of morbid structure, deceived in determining with precision the presence of inflammation or softening. Indeed, how can it be otherwise, when we consider the deceptive nature of the modes in which the investigation is determined? Thus, an intense degree of redness in a tissue is by some called congestion, by others, inflammation. How vague are the ideas attached to the consistence of organs!—what appears healthy to one, seems to another somewhat indurated, and to a third, softened.

On the other hand, we are enabled to determine that lesions supposed to be inflammatory arise solely from hemorrhage, or simple congestion—that what has been considered tubercle was in fact infiltrated pus—that tumors imagined to be malignant, were really innocuous, and so on. Regarding all these points, we are enabled to get rid of the vagueness and looseness which prevail in connection with them, and by means of the microscope, to arrive at positive information, on which the morbid anatomist may safely rely.

The microscope explains to us also why certain diseases are so intractable to treatment. It shows us, that several morbid lesions are composed of cells, each of which possesses an independent vitality. Thus, warts, melanosis, cancer, fungus, hematodes, &c., defy the efforts of the practitioner, because he is unable to attack them through the organism of the individual in whom they exist. In fact, they are distinct beings, endowed with a vitality of their own, true parasites, which feed upon the tissue they are found in, and can only, by its destruction or excision, be removed from the economy.

As it is not possible to become acquainted, by means of naked vision, even with the *healthy* structure of organs, how, therefore, can we expect, by its aid alone, to arrive at the knowledge of *diseased* tissues ? Need we wonder that so many affections and morbid growths are considered incurable, when we remain ignorant of their very nature ?

The good service the microscope has rendered to the cause of science, in such hands as that of the celebrated naturalist, Professor Owen, will be best understood on reference to his writings. His great and valuable work on "Odontography," teems with evidence of this. To give one short extract. He says:—"It has sometimes happened that a few scattered teeth have been the only indications I have been able to find of animal life throughout an extensive stratum; and when these teeth happened not to be characterised by any well marked peculiarity of external form, there remained no other test by which their nature could be ascertained than that of the microscopic examination of their internal tissue. It has settled the doubts entertained by some of the highest authorities in palæontology as to the true affinities of the gigantic megatherium; and by demonstrating the identity of its dental structure with that of the sloth, has yielded us an unerring indication of the true nature of its food." Thus, then, we perceive the study of the teeth presents to the naturalist unbounded objects of interest, and may become the sole disclosers of the past works of creation.

Equal in interest to the physiologist is the search into the minute structures of the teeth, with their delicate cellular arrangement of pulp and its wonderful conversion into ivory and enamel, one of the hardest substances met with in nature. I could not, perhaps, cite a more remarkable conversion to the important disclosures of the microscope than what was related of the late eminent surgeon, Bransby Cooper. He said:—"I must confess that it was with difficulty I could bring my mind to believe, that the investigation of the molecular structure of tissues could ever tend to the advancement of medical science; it required a struggle to overcome the *vis inertiæ* of my mind and the stubbornness of ignorance, before I was induced to examine the minute structures of the various tissues of the human body by the aid of the microscope. I determined, however, to overcome my prejudice, and in a very short time I became impressed with the thorough conviction of the utility of the instrument in all my investigations. By the assistance of this instrument, I for the first time began to understand, that the physical and vital conditions which render the various tissues of the body competent to fulfil their important offices in the animal economy, depend, in a great measure, on the ultimate arrangement of their proximate elements, a knowledge of which could not be acquired by the minutest dissections, unless aided by the microscope. As soon as the structures of the body in a state of health had become by practice easily recognizable under the microscope, I was able to distinguish the smallest portions of bone, cartilage, muscle, or fat, from each other, in consequence of their unvarying structural character, in the normal condition. I was soon incited to examine microscopically the same tissues when affected by disease; and I found that the deviations from their natural oganization, were as appreciable as in their nominal structure; and I am now convinced that the microscope is as necessary to the anatomist and pathologist, as the scalpel to the one, and bedside observation to the other. I, therefore, became satisfied that the smallest portion of diseased structure placed under the microscope, will tell more in one minute to the experienced eye, than could be acquired by a week's examination of the gross masses of disease in the ordinary way." "Thus everything connected with our science sufficiently manifests, that we are now in a much better train than those who preceded us. The wisdom of our ancestors, though highly useful in its day, is fast retiring before the knowledge of the present age, as that which we ourselves may be inclined to boast of may be eclipsed by the labors of the next. Yet, gentlemen, science must advance. The axiom that knowledge is power, is universally received; and, however men, nursed in error, may smile at our enthusiasm, and look coldly on our endeavors, nothing can deter or check the labors of that earnest and united band, whose watchword is the search after and discovery of truth."

I will now, with your permission, devote a short time to the consideration of the construction of the microscope. The term *microscope* is derived from *micros*, little, and *scopio*, to view or see. The microscope may then be defined as an instrument which enables us to view objects which are too *little* to be seen by the unaided or naked eye. Or as an instrument which ena-

Fig. 1.—*The Compound Microscope.*

bles us to see objects distinctly at a shorter distance from the eye than is compatible with distinct vision, (optical angle is here meant,) and thereby enables us to see objects too minute to be seen at the distance of distinct vision. At different distances from the eye, the same object subtends different angles, as may be seen by referring to the diagram, fig. 2. In every lens the right line perpendicular to the two surfaces is called the *axis* of the lens, and is seen in the annexed figure; the

Fig 2

point where the axis cuts the surface is called the *vertex* of the lens; the middle point between them the *centre;* and the distance between them the *diameter.*

Fig. 3.

Fig. 3 is intended to represent the different forms of lenses in use: *a*, is a plane glass of equal thickness throughout; *b*, a meniscus, concave on one side, convex on the other; *c* a double-concave; *d*, a plano-concave; *e*, a double-convex; *f*, a plano-convex.—*Quar. Jour. Dent. Sci.*

## Figures and Tables

**Fig. 1. f1:**
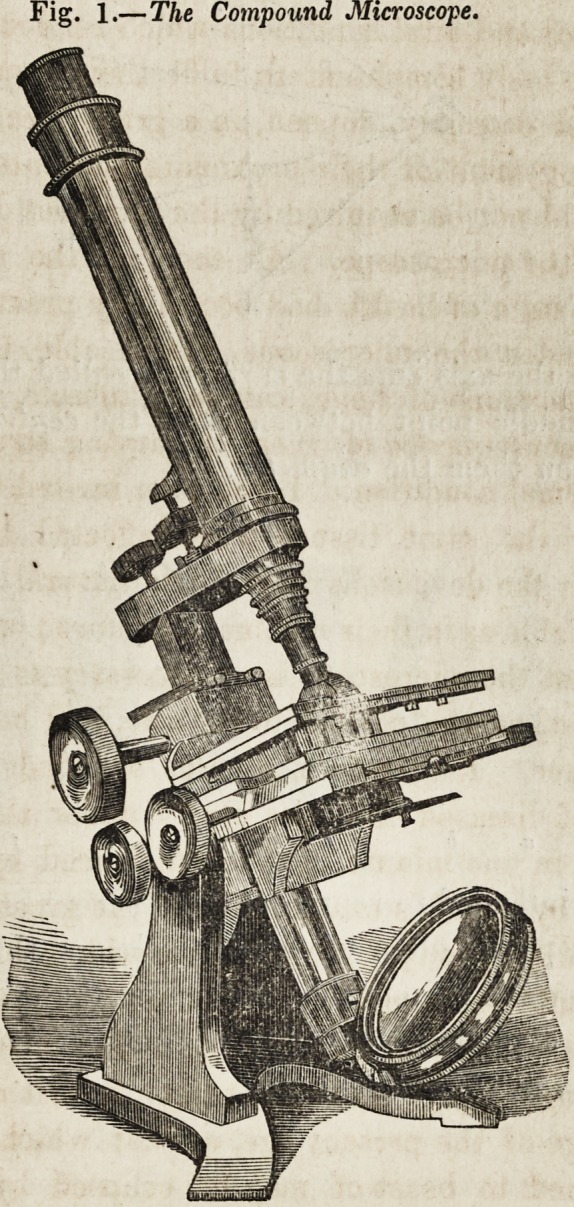


**Fig 2 f2:**
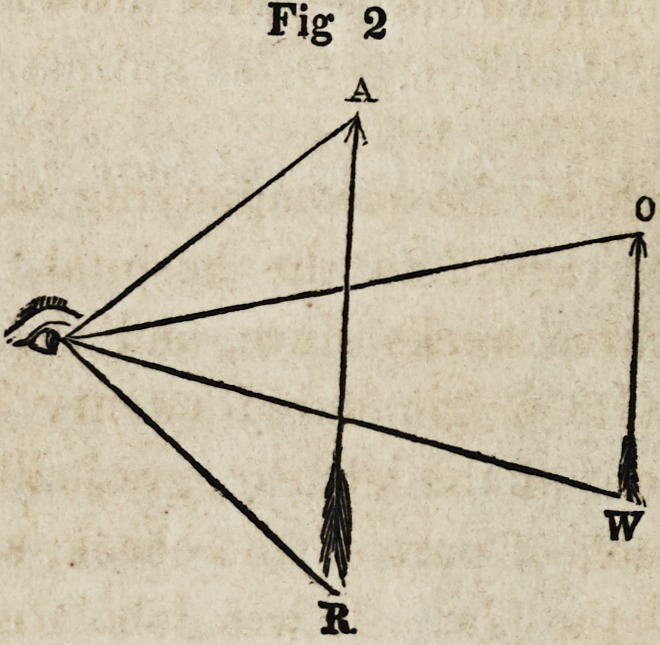


**Fig. 3. f3:**